# Nucleophosmin mutations confer an independent favorable prognostic impact in 869 pediatric patients with acute myeloid leukemia

**DOI:** 10.1038/s41408-019-0268-7

**Published:** 2020-01-09

**Authors:** Lu-Hong Xu, Jian-Pei Fang, Yao-Chung Liu, Adrianna I. Jones, Li Chai

**Affiliations:** 1 Guangdong Provincial Key Laboratory of Malignant Tumor Epigenetics and Gene Regulation, Department of Pediatrics, Sun Yat-Sen Memorial Hospital, Sun Yat-Sen University, Guangzhou, Guangdong Province People’s Republic of China; 2 Department of Pathology, Brigham & Women’s Hospital, Harvard Medical School, Boston, MA 02115 USA; 3 Division of Hematology, Department of Medicine, Taipei Veterans General Hospital, Taipei, Taiwan; School of Medicine, National Yang-Ming University, Taipei, Taiwan; 4 Beth Israel Deaconess Medical Center, Harvard Medical School, Boston, MA 02115 USA; 5 Department of Biology, Davidson College, Davidson, NC 28035 USA

**Keywords:** Acute myeloid leukaemia, Paediatrics

## Abstract

Studies on the clinical significance of Nucleophosmin (NPM1) mutations in pediatric AML in a large cohort are lacking. Moreover, the prognosis of patients with co-occurring NPM1 and FLT3/ITD mutations is controversial. Here, we analyzed the impact of NPM1 mutations on prognoses of 869 pediatric AML patients from the TAGET dataset. The frequency of NPM1 mutations was 7.6%. NPM1 mutations were significantly associated with older age (*P* < 0.001), normal cytogenetics (*P* < 0.001), FLT3/ITD mutations (*P* < 0.001), and high complete remission induction rates (*P* < 0.05). Overall, NPM1-mutated patients had a significantly better 5-year EFS (*P* = 0.001) and OS (*P* = 0.016) compared to NPM1 wild-type patients, and this favorable impact was maintained even in the presence of FLT3/ITD mutations. Stem cell transplantation had no significant effect on the survival of patients with both NPM1 and FLT3/ITD mutations. Multivariate analysis revealed that NPM1 mutations were independent predictors of better outcome in terms of EFS (*P* = 0.004) and OS (*P* = 0.012). Our findings showed that NPM1 mutations confer an independent favorable prognostic impact in pediatric AML despite of FLT3/ITD mutations. In addition, pediatric AML patients with both NPM1 and FLT3/ITD mutations appear to have favorable prognoses and may not need hematopoietic stem cell transplantations.

## Introduction

Acute myeloid leukemia (AML) is a genetically heterogeneous disease that accounts for about 20% of pediatric leukemia. The overall 5-year survival rate for pediatric patients with AML has increased over time and is now in the range of 65–70%^1^. Most of the advances have been made by better risk classification, the implementation of excellent supportive care measures, and improvements in allogeneic hematopoietic stem cell transplantation^2^. However, survival rates still vary depending on the subtype of AML and genetic risk factors. In recent years, molecular analysis has identified novel markers with prognostic relevance.

Nucleophosmin (NPM1) is a widely expressed protein predominantly located in the nucleolus that continuously shuttles between nucleus and cytoplasm. NPM1 performs diverse biological functions including molecular chaperoning, ribosome biogenesis, DNA repair, and maintaining genomic stability^3^. The human NPM1 gene is located on chromosome 5q35 and contains 12 exons. NPM1 mutations cause delocalization of the protein to the cytoplasm and are involved in leukemogenesis^4,5^. The WHO classification of hematopoietic malignancies recognizes AML with mutated NPM1 as a distinct entity^6^. Intriguingly, about 40% of NPM1-mutated AML cases have co-occurring FMS-like tyrosine kinase internal tandem duplication (FLT3/ITD) mutations^7^. However, the prognosis of patients with NPM1 co-occurring FLT3/ITD mutations is controversial. Numerous adult studies have shown that NPM1-mutated patients had improved responses to treatment, only in the absence of FLT3/ITD mutations^8–10^. Clinical data on pediatric AML patients with NPM1 mutations is lacking. A study of 295 childhood AML patients revealed that NPM1 mutations do not abrogate the negative prognostic influence of FLT3/ITD mutations^11^. However, another cohort of 298 childhood AML study showed that NPM1 mutations confer a favorable prognosis in childhood, cytogenetically normal AML regardless of FLT3/ITD mutations^12^. Hence, it is necessary to investigate the clinical significance of NPM1 mutations in pediatric AML in a large cohort.

Here, we analyzed the prevalence of NPM1 mutations in 869 pediatric AML patients from the therapeutically applicable research to generate effective treatment (TARGET) dataset. In addition, we evaluated the impact of these mutations on the patients’ prognoses and clinical profiles. Our findings showed that NPM1 mutations confer an independent favorable prognostic impact in the pediatric AML patients in spite of FLT3/ITD mutations. Moreover, pediatric AML patients with both NPM1 and FLT3/ITD mutations had favorable prognoses and may not require hematopoietic stem cell transplantations.

## Materials and methods

### Clinical data on pediatric patients with AML

Complete clinical data for 869 pediatric AML patients younger than 18 years old was downloaded from the TARGET dataset (April 2, 2019) (https://ocg.cancer.gov/programs/target/data-matrix). According to the dataset, year of diagnosis ranged from 1996 to 2010. Year of last follow-up ranged from 1997 to 2015. Diagnosis and subtype classifications of AML were assigned according to the French–American–British (FAB) classifications. Treatment protocols for AML included AAML03P1, AAML0531 and CCG-2961, all of which consisted of a remission induction phase followed by an intensification phase. Stem cell transplantation (SCT) was considered for patients in the first complete remission. Written informed consent was obtained from all study participants. Informed consent was obtained in accordance with the Declaration of Helsinki. Cytogenetic analyses in situ were performed by standard G-banding/fluorescence in situ hybridization techniques. Molecular analyses for FLT3/ITD and NPM1 mutations were performed on genomic DNA by polymerase chain reaction (PCR).

### Statistical analysis

Chi-squared analysis and Fisher’s exact test, in cases of small numbers, were used to compare categorical variables. The nonparametric Mann–Whitney *U*-test was applied for continuous variables. To assess outcome, the following parameters were used: complete remission (CR, defined as a normocellular BM containing fewer than 5% blasts) rate, event-free survival (EFS, defined as time between diagnosis and first event, including induction failure, relapse or death of any cause), overall survival (OS, defined as time between diagnosis and death from any cause). EFS and OS were estimated by the Kaplan–Meier method and compared using the log-rank test. Prognostic factors were examined by multivariate Cox regression analysis. *P*-values of <0.05 were considered statistically significant (two-tailed testing). The data were analyzed with the Statistical Package for the Social Sciences (SPSS®) version, 24.0 (IBM Corporation, Armonk, NY, USA).

## Results

### **Relationship between NPM1 mutations and clinical characteristics**

The characteristics of the study population, according to NPM1 mutation status, are shown in Table 1. Totally, among the 869 pediatric patients with AML, 66 patients (7.6%) were identified with NPM1 mutations. The median age in NPM1 mutations group and NPM1 wild-type group were 13.4 and 9.1, respectively, and the differences were statistically significant (*P* < 0.001). Moreover, the frequency was increasing along with the age in NPM1 mutations group: 4.5% below the age of 3 years; 22.7% in the age group 3 years or older but below 10 years, and 72.7% in children aged 10 years or older.

**Table 1:** Characteristics of study population according to NPM1 mutation status.

	All patients	NPM1-mutated case	NPM1 wild-type case	*P*-value
Number (%)	869	66 (7.6%)	803 (92.4%)	
Age, median (year)	9.6	13.4	9.1	<0.001
<3 years, *n* (%)	211 (24.3%)	3 (4.5%)	208 (25.9%)	<0.001
3 ≤ Age <10 years, *n* (%)	237 (27.3%)	15 (22.7%)	222 (27.6%)	0.388
10 ≤ Age <18 years, *n* (%)	421 (48.4%)	48 (72.7%)	373 (46.5%)	<0.001
Sex (% female)	47.6%	50%	47.4%	0.690
WBC, × 10^9^/L, Median (rang)	31.7 (0.2-610)	28.6 (2.1-360.5)	32.1 (0.2-610)	0.541
FAB classification: *N* (%)				0.151
M0	20 (2.8%)	0 (0%)	20 (3.0%)	0.393
M1	96 (13.5%)	15 (26.8%)	81 (12.3%)	0.002
M2	193 (27.1%)	13 (23.2%)	180 (27.4%)	0.499
M3	2 (0.3%)	1 (1.8%)	1 (0.2%)	0.151
M4	192 (26.9%)	11 (19.6%)	181 (27.5%)	0.200
M5	160 (22.4%)	16 (28.6%)	144 (21.9%)	0.252
M6	11 (1.5%)	0 (0%)	11 (1.7%)	>0.999
M7	39 (5.5%)	0 (0%)	39 (5.9%)	0.064
FLT3/ITD				<0.001
Positive, *n* (%)	146 (16.8%)	24 (36.4%)	122 (15.2%)	
Negative, *n* (%)	722 (83.2%)	42(63.6%)	680 (84.8%)	
FLT3/ITD allelic ratio				
Median (rangE)	0.54 (0.03-9.50)	0.48 (0.03-9.50)	0.55 (0.03-5.19)	0.551
Cytogenetic status				<0.001
Normal (*n*, %)	196 (23.7%)	51 (81.0%)	145 (19.0%)	
Abnormal (*n*, %)	631 (76.3%)	12 (19.0%)	619 (81.0%)	
SCT in 1st CR				0.001
No (*n*, %)	661 (83.7%)	45 (69.2%)	616 (85.0%)	
Yes (*n*, %)	129 (16.3%)	20 (30.8%)	109 (15.0%)	
Protocol				0.915
AAML03P1 (*n*, %)	91 (10.5%)	6 (9.1%)	85 (10.6%)	0.703
AAML0531 (*n*, %)	732 (84.2%)	56 (84.8%)	676 (84.2%)	0.887
CCG-2961 (*n*, %)	46 (5.3%)	4 (6.1%)	42 (5.2%)	0.772
CR status at end of course 1				0.022
CR	655 (76.3%)	57 (87.7%)	598 (75.3%)	0.024
Not CR	189 (22.0%)	8 (12.3%)	181 (22.8%)	0.050
Death	15 (1.7%)	0 (0%)	15 (1.9%)	0.620
CR status at end of course 2				0.014
CR	735 (87.2%)	63 (96.9%)	672 (86.4%)	0.015
Not CR	88 (10.4%)	2 (3.1%)	86 (11.1%)	0.043
Death	20 (2.4%)	0 (0%)	20 (2.6%)	0.392

*CR* complete remission, *FAB* French–American–British morphology classification, *FLT3/ITD* internal tandem duplication of the *FLT3* gene, *SCT* stem cell transplantation, *WBC* white blood cell count

The distribution of FAB subtypes in NPM1 mutations group were mainly in M1, M2, M4, and M5 subgroups. Compared with the NPM1 wild-type group, the percentage of M1 subgroup was higher in NPM1 mutations group (26.8% vs 12.3%, *P* = 0.002). The treatment protocols for pediatric AML were equally distributed between these two groups (*P* = 0.915). Notably, NPM1 mutations were significantly associated with FLT3/ITD mutations (*P* < 0.001), normal cytogenetics (*P* < 0.001), transplantation status (*P* = 0.001). However, no significant difference was found in the median of FLT3/ITD allelic ratio between NPM1 mutations group and NPM1 wild-type group (*P* = 0.551).

### Prognostic impact of NPM1 and FLT3/ITD mutations in pediatric AML

The CR rate was determined for the pediatric patients with AML. At the end of the first course of therapy, 57 (87.7%) of the 65 patients with NPM1 mutations achieved a CR compared with 598 (75.3%) of 794 patients without NPM mutations (*P* = 0.024). At the end of the second course of therapy, 63 (96.9%) of the 65 patients with NPM1 mutations achieved a CR compared with 672 (86.4%) of 778 patients without NPM mutations (*P* = 0.015). Thus, NPM1 mutations were significantly associated with high induction CR rates.

**Fig. 1:**
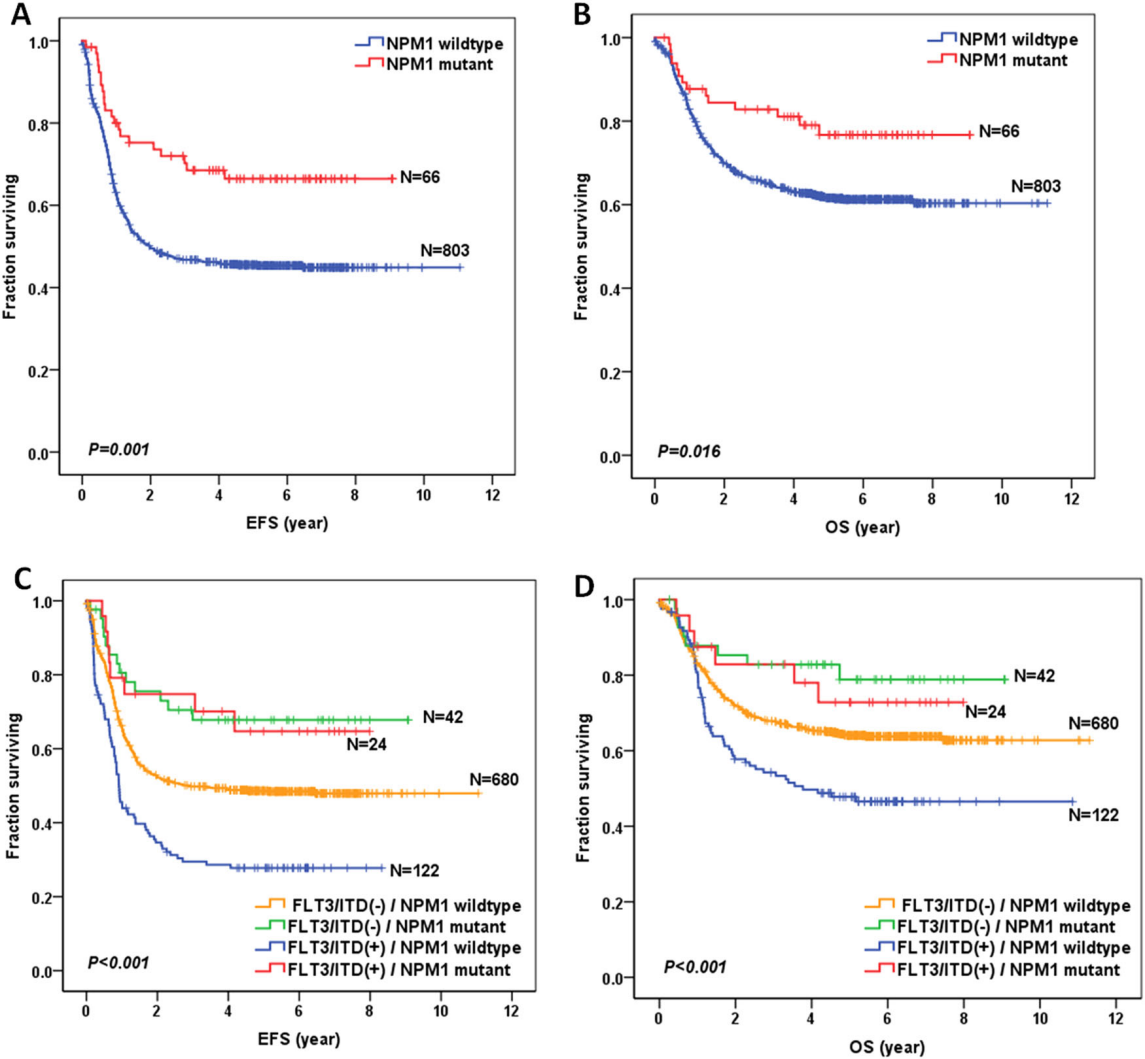
Survival curves of all pediatric AML patients with and without NPM1 mutations, and according to the combined NPM1 and FLT3/ITD status. **a** Probability of EFS for patients with and without NPM1 mutations. **b** Probability of OS for patients with and without NPM1 mutations. **c** Probability of EFS for patients according to the combined NPM1 and FLT3/ITD status. **d** Probability of OS for patients according to the combined NPM1 and FLT3/ITD status.

We then evaluated the survival data for all the 869 pediatric patients. The median follow-up time for all the survivors was 5.6 years. As shown in Fig. 1a, NPM1-mutated patients had a significantly better 5-year EFS (66.4 ± 6.0%) compared to wild-type patients (45.5 ± 1.8%; *P* = 0.001). Moreover, NPM1-mutated patients had a significantly better 5-year OS (76.7 ± 5.6%) compared with wild-type patients (61.6 ± 1.8%; *P* = 0.016) (Fig. 1b).

Survival data were also examined for the 868 patients for whom the FLT3/ITD mutation status was known (Fig. S1a, b). The presence of a FLT3/ITD mutation was significantly associated with inferior EFS (5-year EFS = 33.7 ± 4.0% vs 49.7 ± 1.9% for FLT3/ITD-negative; *P* < 0.001) and OS (5-year EFS = 51.9 ± 4.3% vs 64.9 ± 1.8% for FLT3/ITD-negative; *P* = 0.004). Next, we further investigated the effect of FLT3/ITD allelic ratio (AR) on survival using an AR threshold of 0.4 (Fig. S1c, d). However, we found that FLT3/ITD AR yielded no significant differences in 5-year EFS (30.9 ± 4.8% vs 38.5 ± 7.1%; *P* = 0.097) and OS (50.5 ± 5.3% vs 54.1 ± 7.3%; *P* = 0.567) between pediatric patients with high AR (>0.4, *n* = 95) and low AR (≤0.4, *n* = 51).

Since there is a significant association between NPM1 and FLT3/ITD status, subgroup analysis was performed to assess the relative contributions of NPM1 and FLT3/ITD to patient prognosis (Fig. 1c, d; Table 2). FLT3/ITD was a significantly poor prognostic factor for the NPM1 wild-type AML patients. However, we did not find that FLT3/ITD mutations, in combination with NPM1 mutations, had a negative influence on patient outcome (EFS hazard ratio: 1.050 (0.435–2.534), *P* = 0.914; OS hazard ratio: 1.283 (0.445–3.698), *P* = 0.645). Notably, when restricted to the FLT3/ITD-positive subgroup, NPM1-mutated patients had improved EFS (5-year EFS = 64.7 ± 10.2% vs 27.8 ± 4.1% for NPM1 wild-type patients; hazard ratio: 0.323 [0.156–0.667], *P* = 0.002) and OS (5-year OS = 72.8 ± 9.6% vs 47.8 ± 4.7% for NPM1 wild-type patients; hazard ratio: 0.408 [0.176–0.944], *P* = 0.022).

**Table 2:** Statistical comparison of survival data according to both NPM1 and FLT3/ITD status in 868 pediatric AML.

Comparison	EFS hazard ratio (95% CI)	EFS *P*-value	OS hazard ratio (95% CI)	OS *P*-value
FLT3/ITD(−): NPM1 wild-type vs NPM1 mutant	0.524 (0.301–0.912)	0.022	0.509 (0.251–1.029)	0.060
FLT3/ITD(+): NPM1 wild-type vs NPM1 mutant	0.323 (0.156–0.667)	0.002	0.408 (0.176–0.944)	0.036
NPM1 wild-type: FLT3/ITD(−) vs FLT3/ITD(+)	1.763 (1.393–2.231)	<0.001	1.612 (1.218–2.132)	0.001
NPM1 mutant: FLT3/ITD(−) vs FLT3/ITD(+)	1.050 (0.435–2.534)	0.914	1.283 (0.445–3.698)	0.645

*CI* confidence interval, *EFS* event-free survival, *FLT3/ITD* internal tandem duplication of the FLT3 gene, *OS* overall survival

### Prognostic impact of NPM1 mutations in the subgroup of cytogenetically normal AML

**Fig. 2:**
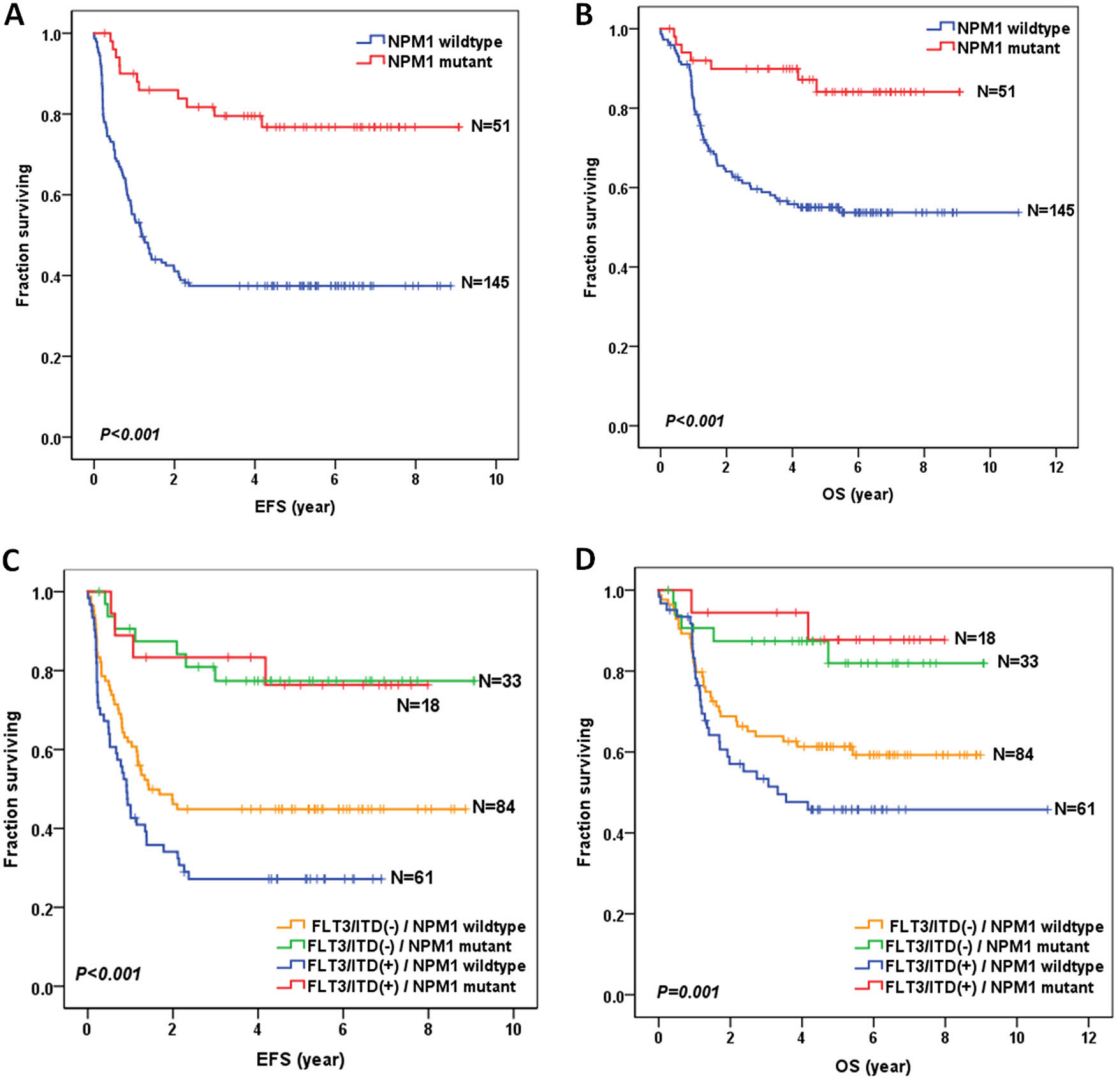
Survival curves of the subgroup of cytogenetically normal AML patients with and without NPM1 mutations, and according to the combined NPM1 and FLT3/ITD status. **a** Probability of EFS for patients with and without NPM1 mutations. **b** Probability of OS for patients with and without NPM1 mutations. **c** Probability of EFS for patients according to the combined NPM1 and FLT3/ITD status. **d** Probability of OS for patients according to the combined NPM1 and FLT3/ITD status.

Overall, 196 pediatric patients were restricted to the subgroup of cytogenetically normal AML. As shown in Fig. 2a, b, NPM1 mutations conferred a favorable impact in prognosis in the subgroup of cytogenetically normal AML. The 5-year EFS for NPM1-mutated patients (*n* = 51) and NPM1 wild-type patients (*n* = 145) were 76.8 ± 6.2% and 37.4 ± 4.1%, respectively, and the difference was statistically significant (*P* < 0.001). In addition, the 5-year OS for these two groups were 84.1 ± 5.7% and 55.0 ± 4.2%, respectively (*P* < 0.001).

The data for combined NPM1 and FLT3/ITD status in the subgroup of cytogenetically normal AML are shown in Fig. 2c, d. Notably, there were no survival differences between NPM1-mutated-FLT3/ITD-positive (*n* = 18) and NPM1-mutated-FLT3/ITD-negative patients (*n* = 33), in terms of 5-year EFS (76.4 ± 10.4% vs 77.4 ± 7.5%; *P* = 0.985) and OS (87.7 ± 8.2% vs 81.9 ± 7.7%; *P* = 0.585).

### Prognostic impact of NPM1 and SCT in pediatric AML

**Fig. 3:**
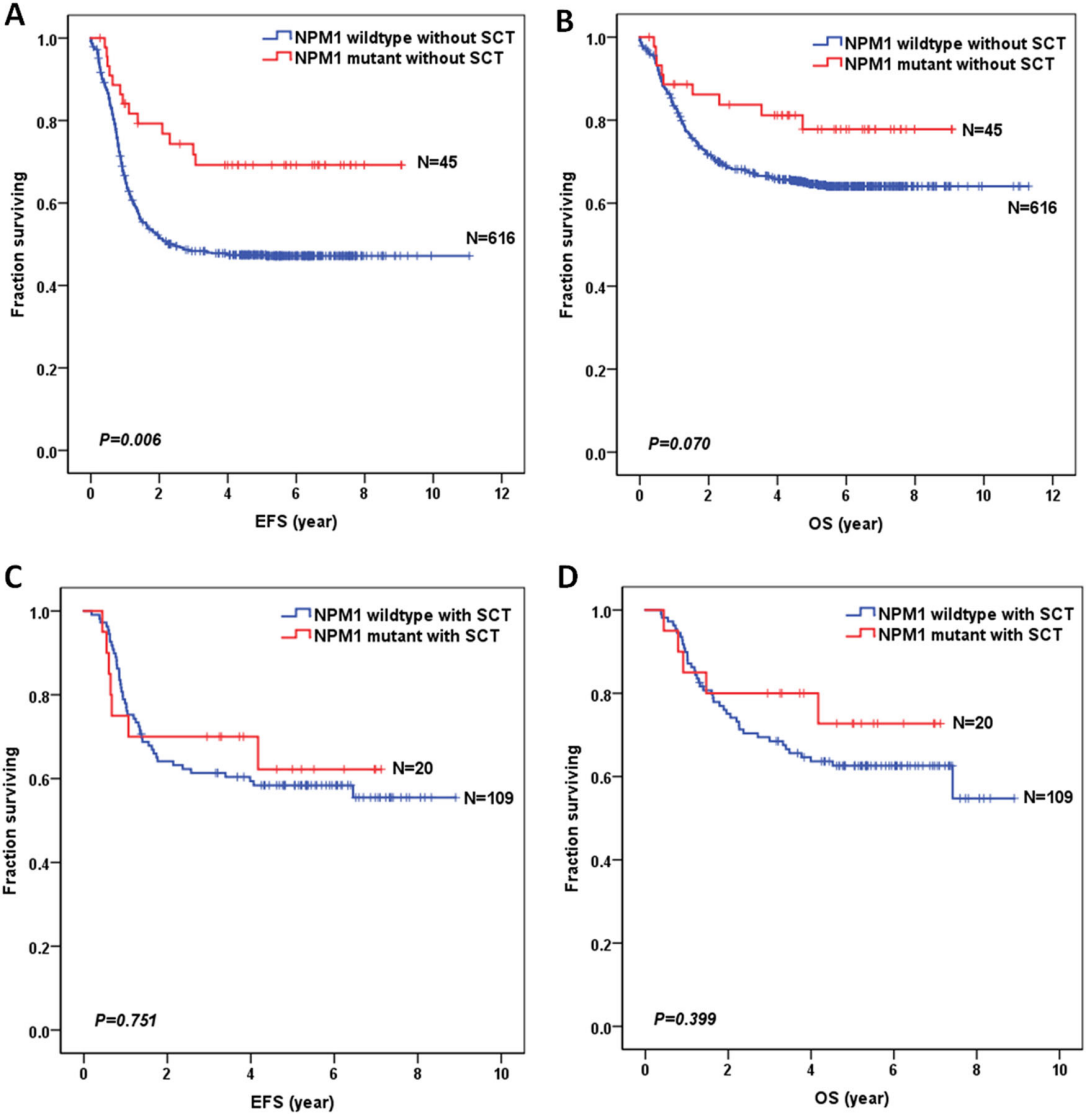
Survival curves of all pediatric AML patients according to the combined NPM1 and SCT status. **a** Probability of EFS for patients without SCT. **b** Probability of OS for patients without SCT. **c** Probability of EFS for patients with SCT. **d** Probability of OS for patients with SCT.

The percentage of SCT in NPM1 mutations group was higher than that in NPM1 wild-type group (30.8% vs 15.0%, *P* = 0.001). The survival analysis, after SCT stratification, of 790 NPM1-mutated pediatric AML patients is shown in Fig. 3. When restricted to no SCT, NPM1 mutations conferred a favorable prognostic impact on 5-year EFS (69.2 ± 7.1% vs 47.4 ± 2.0%; *P* = 0.006) and a trend of better 5-year OS (77.8 ± 6.6% vs 64.6 ± 2.0%; *P* = 0.07). However, when restricted to SCT, there was no significant difference between the NPM1-mutated group and the NPM1 wild-type group in terms of 5-year EFS (*P* = 0.751) and OS (*P* = 0.399).

**Fig. 4:**
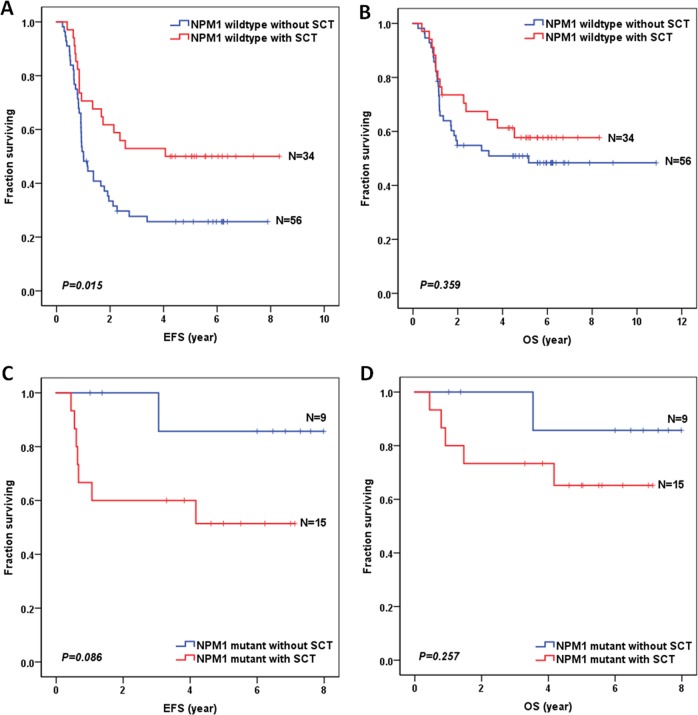
Survival curves of FLT3/ITD-positive patients excluded with induction failure or death without complete remission, according to the combined NPM1 and SCT status. **a** Probability of EFS for patients with NPM1 wild-type and FLT3/ITD mutations. **b** Probability of OS for patients with NPM1 wild-type and FLT3/ITD mutations. **c** Probability of EFS for patients with both NPM1 and FLT3/ITD mutations. **d** Probability of OS for patients with both NPM1 and FLT3/ITD mutations.

In addition, to evaluate the role of SCT in AML patients according to FLT3/ITD status, we excluded 34 cases of AML patients with induction failure or death without CR in the analyses. As shown in Fig.S2 and Table S1, SCT appeared to improve 5-year EFS for patients with FLT3/ITD-negative (65.4 ± 5.4% vs 53.4 ± 2.1% for patients without SCT; hazard ratio: 0.672 [0.455-0.993], *P* = 0.046) or FLT3/ITD-positive (50.7 ± 7.2% vs 33.4 ± 6.0% for patients without SCT; hazard ratio: 0.628 [0.380–1.040], *P* = 0.070), but the differences were not statistically significant in FLT3/ITD-positive group. To further evaluate the role of SCT in patients with NPM1 co-occurring FLT3/ITD mutations, we excluded seven cases of FLT3/ITD-positive patients with induction failure or death without CR in the analyses. As shown in Fig. 4a, b, SCT improved the survival of patients with NPM1 wild-type and FLT3/ITD mutations in term of 5-year EFS (50.0 ± 8.6% vs 25.7 ± 5.9% for patients without SCT, *P* = 0.015), which did not translate into a significantly better 5-year OS (57.7 ± 8.7% vs 50.9 ± 6.8% for patients without SCT, *P* = 0.359). However, SCT had no significant effect on the survival of patients with both NPM1 and FLT3/ITD mutations, in term of 5 year-EFS (51.4 ± 13.4% vs 85.7 ± 13.2% for patients without SCT, *P* = 0.086) or OS (65.2 ± 12.7% vs 85.7 ± 13.2% for patients without SCT, *P* = 0.257) (Fig. 4c, d).

### Multivariate analysis of prognostic factors

When we included NPM1 mutations with other risk factors in the Cox model, including age (utilizing 10 years of age as the cutoff value), abnormal cytogenetics, FLT3/ITD and SCT as co-variables, we identified NPM1 mutations as an independent factor for both EFS and OS in pediatric patients with AML (Table 3). NPM1 mutations were significantly associated with better EFS (HR: 0.473, 95% CI: 0.283–0.790, *P* = 0.004) and OS (HR: 0.452, 95% CI: 0.242–0.841, *P* = 0.012). Moreover, SCT was significantly associated with better EFS (HR: 0.575, 95% CI: 0.419–0.790, *P* = 0.001), but not with better OS (HR: 0.824, 95% CI: 0.580–1.171, *P* = 0.280). On the contrary, FLT3/ITD mutations were significantly associated with worse EFS (HR: 1.743, 95% CI: 1.296–2.345, *P* < 0.001) and OS (HR: 1.600, 95% CI: 1.132–2.263, *P* = 0.008). In this model, the age and abnormal cytogenetics did not reach significance either for EFS or for OS.

**Table 3:** Multivariate analysis for EFS and OS in pediatric patients with AML.

Outcome	Variable	Hazard ratio (95% CI)	*P*-value
EFS	NPM1	0.473 (0.283–0.790)	0.004
	FLT3/ITD	1.743 (1.296–2.345)	<0.001
	SCT	0.575 (0.419–0.790)	0.001
	Age >10	1.098 (0.889–1.356)	0.387
	Abnormal cytogenetics	1.058 (0.789–1.420)	0.705
OS	NPM1	0.452 (0.242–0.841)	0.012
	FLT3/ITD	1.600 (1.132–2.263)	0.008
	SCT	0.824 (0.580–1.171)	0.280
	Age >10	1.192 (0.924–1.538)	0.176
	Abnormal cytogenetics	0.995 (0.707–1.401)	0.977

*CI* confidence interval, *EFS* event-free survival, *FLT3/ITD* internal tandem duplication of the FLT3 gene, *OS* overall survival, *SCT* stem cell transplantation

## Discussion

The TARGET program applies a comprehensive genomic approach to determine molecular changes that drive childhood cancers. The TARGET AML project team consists of multiple Children’s Oncology Group (COG) investigators at various institutions. The frequency of NPM1 mutations among 869 pediatric AML was 7.6%, which was lower compared with that of adult AML (range 25.4–41%). In agreement with other studies, NPM1 mutations have an increased incidence with increasing age. Three cases of NPM1 mutations were found in children below the age of 3 years in our study. However, no NPM1 mutations were found in children of this age group in other studies. Moreover, NPM1 mutations were predominately found in the FAB subtypes of M1, M2, M4, and M5. In other studies, however, the M5 subgroup harbored no NPM1 mutations^12,13^. The differences might be due to our study including a large serial of pediatric patients. Our study confirmed that NPM1 mutations were significantly associated with FLT3/ITD mutations and normal cytogenetics. In addition, among the NPM1-mutated patients, 19% (12/63) of patients were identified with abnormal cytogenetics (Table S2). Only one NPM1-mutated patient was found either with the favorable karyotype t(8;21) or inv(16).

In agreement with adult studies^8,14^, NPM1 mutations were significantly associated with high induction CR rates in our study. However, another pediatric study showed that NPM1 mutation status did not significantly affect induction CR rate^11^. In our study of 869 pediatric AML patients, we showed an independent favorable outcome for the patients with NPM1 mutations in terms of 5-year EFS (*P* = 0.001) and OS (*P* = 0.016) compared to the patients with NPM1 wild-type. In addition, when focusing on the cytogenetically normal subgroup, NPM1 mutations also yielded better 5-year EFS (*P* < 0.001) and OS (*P* < 0.001) compared to NPM1 wild-type patients. However, when focusing on the cytogenetically abnormal subgroup, NPM1 mutations had no effect on EFS (*P* = 0.159) and OS (*P* = 0.556) (Fig. S3a, b). Furthermore, when excluding either an inv(16) or t(8; 21) in the cytogenetically abnormal subgroup, NPM1 mutations did not show favorable impact on EFS (*P* = 0.411) or OS (*P* = 0.798) in those patients (Fig. S3c, d). These findings were different from an adult cohort study, which showed that NPM1 mutations conferred favorable prognosis independently of whether cytogenetical status was normal or not^15^.

It has been reported, in both adult and pediatric studies, that FLT3/ITD-mutated AML patients have poor prognoses^16,17^. Furthermore, patients with a high AR of FLT3/ITD to wild-type alleles were reported to show poor prognosis in pediatric AML^18,19^. In our study, we found that FLT3/ITD mutations were significantly associated with poor EFS and OS. However, using an AR of 0.4, as described in a pediatric AML report^18^, FLT3/ITD mutations yielded no significant differences in survival between high AR cases and low AR cases. Moreover, we did not find a negative influence on the outcome of FLT3/ITD mutations in the NPM1-mutated patients. Notably, within the FLT3/ITD-positive subgroup, NPM1-mutated patients had improved EFS and OS. NPM1 mutations confer an independent favorable impact in pediatric patients in spite of FLT3/ITD mutations. These findings were consistent with a pediatric study of Hollink et al.^12^. A large cohort of young adult patients with AML also reported that the beneficial impact of NPM1 mutations on survival was seen in FLT3/ITD-positive as well as FLT3 wild-type patients^20^. Another adult study showed that effect of FLT3/ITD burden was modulated by NPM1 mutation, especially in patients with a low ratio^21^. The differences between adult and pediatric studies are worthy of further investigation.

Next, we investigated the effect of SCT in pediatric patients with AML. We found that NPM1 mutations conferred favorable prognostic impact on survival in patients without SCT. SCT improved the EFS of patients with NPM1 wild-type and FLT3/ITD mutations. These findings are consistent with the concept that SCT is indicated for FLT3/ITD-positive AML patients without NPM1 mutations^22^. However, our study revealed that SCT had no significant effect on the survival of patients with both NPM1 and FLT3/ITD mutations. Our results indicate that pediatric AML patients with both NPM1 and FLT3/ITD mutations had favorable prognoses and may not require hematopoietic stem cell transplantations. Multivariate analysis revealed that both NPM1 and FLT3/ITD mutations were highly significant independent predictors of outcome, while SCT was significantly associated with better EFS but not with better OS. The role of SCT in AML patients with both NPM1 and FLT3/ITD mutations remains controversial. According to the 2017 European LeukemiaNet recommendations, FLT3/ITD-positive AML patients with NPM1 mutations are not a priori assigned to allogeneic SCT in first CR^23^. However, some studies indicated that allogeneic SCT improves the prognosis in NPM1-mutated AML with FLT3/ITD low AR^24,25^. Recently, Huang et al.^26^ found that SCT have better survival in adult AML patients with both NPM1 and FLT3/ITD mutations comparing to chemotherapy alone.

The mechanism of NPM1 mutation in leukemogenesis remains unclear. Frameshift mutations in exon 12 of NPM1 are the most common mutation. All of the variants result in the insertion of four base pairs in the C-terminal region, causing loss of a nucleolar localization signal and aberrant localization of the protein to the cytoplasm. The types of NPM1 mutations are different between adult AML and pediatric AML. Type A mutation accounts for ~80% of all variants in adults but only accounts for 11.1–50% of all variants in pediatrics^27^. Hollink et al.^12^ found that no significant differences in outcome were detected between the different types of NPM1 mutations in pediatric AML. These findings were confirmed by some adult AML studies^28,29^. However, Alpermann et al.^30^ found that patients with the type-A mutation had less overall survival and worse event-free survival when compared to those harboring other NPM1 mutations. Moreover, their study showed that FLT3/ITD mutations in combination with the type-A mutation have much poorer prognosis when compared to FLT3/ITD mutations with type B and D cases. Recently, Patel et al.^31,32^ have reported that high variant allele frequency of NPM1 predict poor outcomes in de novo AML and the effect is not affected by FLT/ITD. Murine model studies have showed that mutant NPM1 and FLT3/ITD exhibit a marked and potent molecular synergy toward driving AML pathogenesis^33,34^. The mechanistic links between the various NPM1 mutations and FLT3/ITD reported in various clinical studies need to be further explored in molecular and murine model studies.

Taken together, we analyzed the impact of NPM1 mutations in 869 patients with AML, which is the largest pediatric AML cohort studied to date. Our findings showed that NPM1 mutations confer an independent favorable prognostic impact in pediatric AML, particularly in cytogenetically normal AML cases. Moreover, NPM1 mutations might be able to abrogate the negative prognostic influence of FLT3/ITD mutations. Pediatric patients with both NPM1 and FLT3/ITD mutations might have favorable prognoses, and those patients might not be requiring hematopoietic stem cell transplantation. Well-designed prospective studies are needed to evaluate the impact of hematopoietic stem cell transplantation in pediatric AML with co-occurring NPM1 and FLT3/ITD mutations.

## Supplementary information

Supplementary figure legends

Figure S1

Figure S2

Figure S3

Table S1

Table S2
