# Supplementary material for: Nucleophosmin mutations confer an independent favorable prognostic impact in 869 pediatric patients with acute myeloid leukemia
Source: Blood Cancer J. 2020 Jan 9;10(1):1. doi: 10.1038/s41408-019-0268-7 (PMC6949268; doi:10.1038/s41408-019-0268-7)
Supplement: Supplementary file 1 — Supplementary figure legends [file 41408_2019_268_MOESM1_ESM.docx]

**Supplementary figure legends**

**Fig. S1** Survival curves of pediatric AML patients according to FLT3/ITD status, and according to the level of FLT3/ITD allelic ratio (AR). **a** Probability of EFS for patients according to FLT3/ITD status. **b** Probability of OS for patients according to FLT3/ITD status. **c** Probability of EFS for patients according to the level of FLT3/ITD AR. **d** Probability of OS for patients according to the level of FLT3/ITD AR.

**Fig. S2** Survival curves of 755 pediatric AML patients excluded with induction failure or death without complete remission, according to the combined FLT3/ITD and SCT status. **a** Probability of EFS. **b** Probability of OS.

**Fig. S3** Survival curves of abnormal cytogenetic AML patients according to NPM1 status.  **a** Probability of EFS for abnormal cytogenetic patients. **b** Probability of OS for abnormal cytogenetic patients. **c** Probability of EFS for abnormal cytogenetic patients excluded with either an inv(16) or t (8; 21).  **d** Probability OS for abnormal cytogenetic patients excluded with either an inv(16) or t (8; 21).
