# Supplementary figures and images for: Nucleophosmin mutations confer an independent favorable prognostic impact in 869 pediatric patients with acute myeloid leukemia
Source: Blood Cancer J. 2020 Jan 9;10(1):1. doi: 10.1038/s41408-019-0268-7 (PMC6949268; doi:10.1038/s41408-019-0268-7)

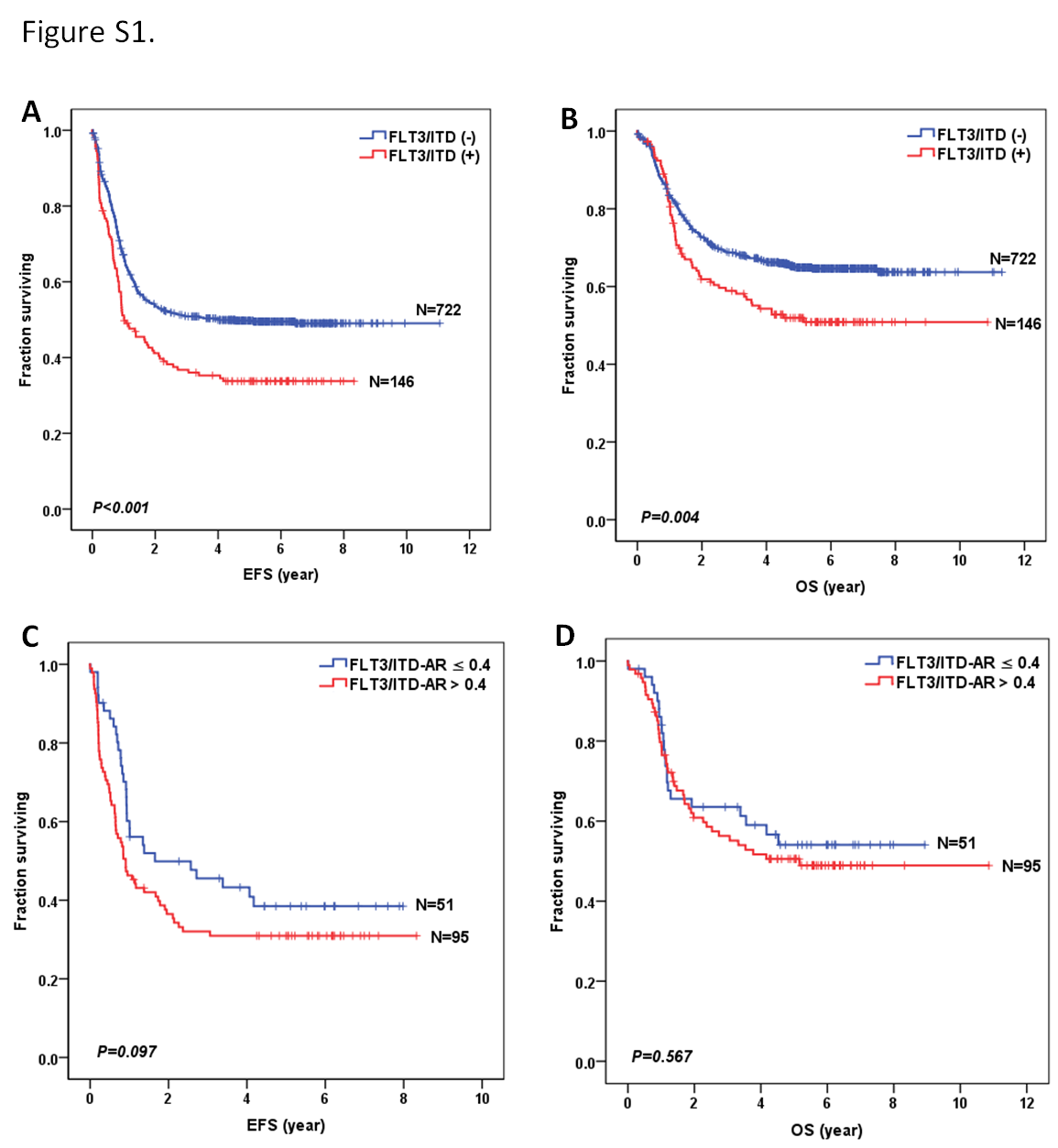

Supplement: Supplementary file 2 — Figure S1 [file 41408_2019_268_MOESM2_ESM.tif]

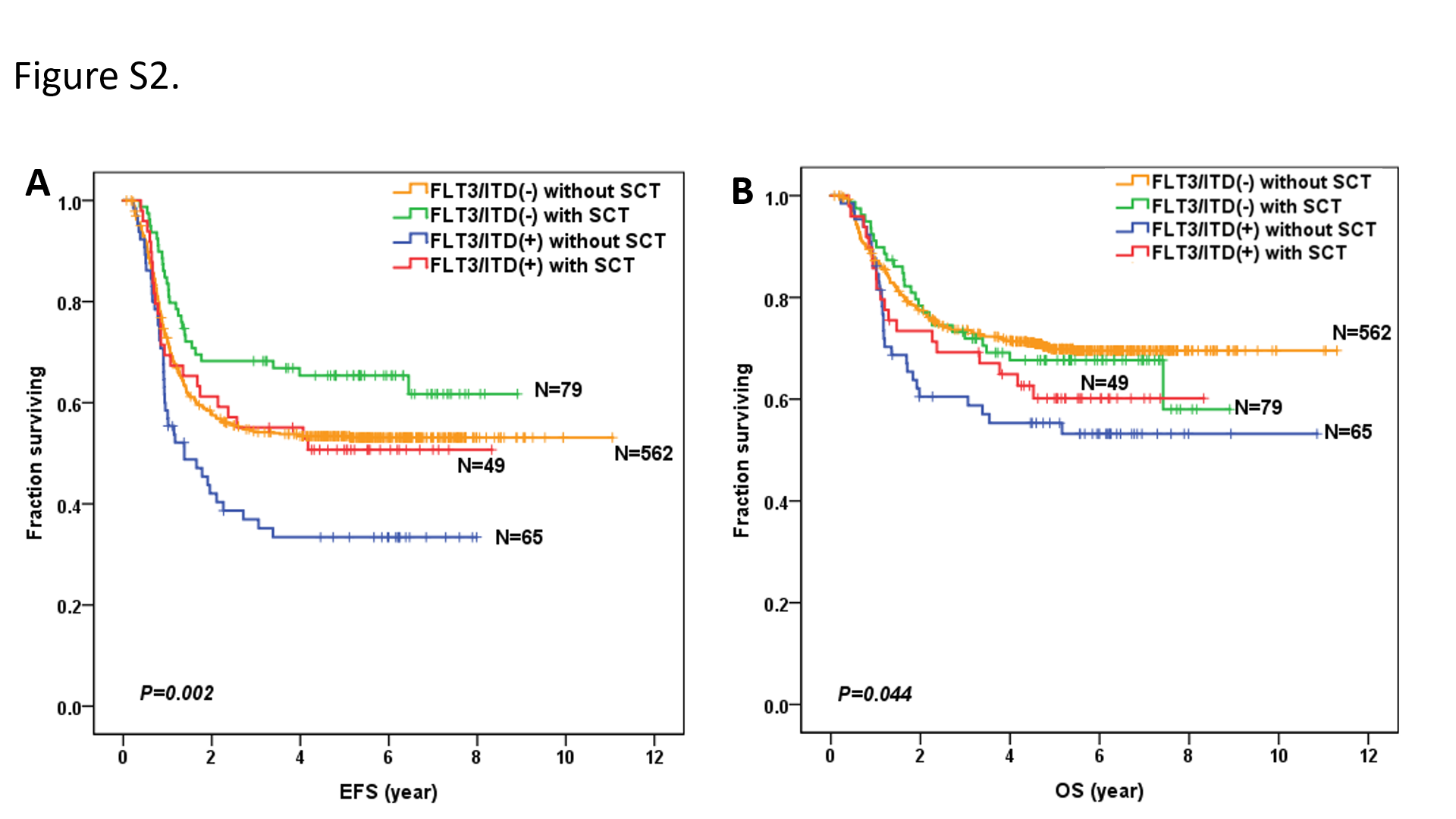

Supplement: Supplementary file 3 — Figure S2 [file 41408_2019_268_MOESM3_ESM.tif]

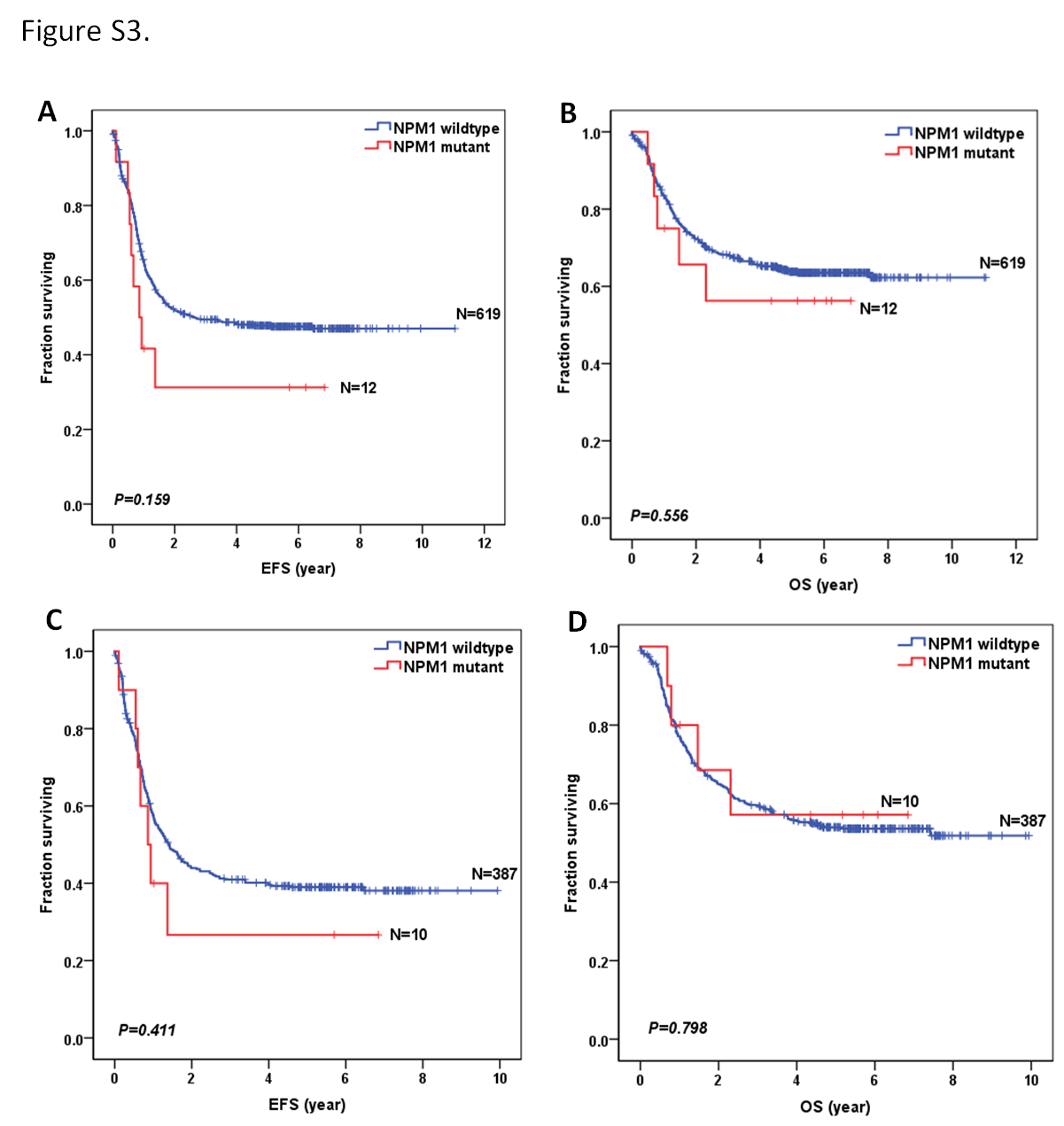

Supplement: Supplementary file 4 — Figure S3 [file 41408_2019_268_MOESM4_ESM.tif]
