# Supplementary material for: Nucleophosmin mutations confer an independent favorable prognostic impact in 869 pediatric patients with acute myeloid leukemia
Source: Blood Cancer J. 2020 Jan 9;10(1):1. doi: 10.1038/s41408-019-0268-7 (PMC6949268; doi:10.1038/s41408-019-0268-7)
Supplement: Supplementary file 5 — Table S1 [file 41408_2019_268_MOESM5_ESM.docx]

Table S1. Statistical comparison of survival data according to both FLT3/ITD and SCT status in 755 pediatric AML

| Comparison | EFS hazard ratio  (95% CI) | EFS  *P*-value | OS hazard ratio  (95% CI) | OS  *P*-value |
| --- | --- | --- | --- | --- |
| FLT3/ITD (-): without SCT vs with SCT | 0.672 (0.455-0.993) | 0.046 | 1.085 (0.718-1.641) | 0.698 |
| FLT3/ITD (+): without SCT vs with SCT | 0.628 (0.380-1.040) | 0.070 | 0.809 (0.453-1.444) | 0.474 |

Abbreviations: CI, confidence interval; EFS, event-free survival; FLT3/ITD, internal tandem duplication of the FLT3 gene; OS, overall survival; SCT, stem cell transplantation.
