# Supplementary material for: Nucleophosmin mutations confer an independent favorable prognostic impact in 869 pediatric patients with acute myeloid leukemia
Source: Blood Cancer J. 2020 Jan 9;10(1):1. doi: 10.1038/s41408-019-0268-7 (PMC6949268; doi:10.1038/s41408-019-0268-7)
Supplement: Supplementary file 6 — Table S2 [file 41408_2019_268_MOESM6_ESM.docx]

Table S2. Differences in the frequency of abnormal cytogenetics according to NPM1 mutation status

|  | NPM1-mutated case  (n=12) | NPM1 wild-type case  (n=619) |
| --- | --- | --- |
| inv(16) | 1 | 104 |
| t (8; 21) | 1 | 128 |
| t (6; 9) | 0 | 17 |
| del5q | 0 | 8 |
| del7q | 0 | 22 |
| del9q | 2 | 31 |
| monosomy 5 | 0 | 1 |
| monosomy 7 | 0 | 18 |
| trisomy 8 | 3 | 81 |
| trisomy 21 | 0 | 23 |
| MLL | 1 | 124 |
| Other | 4 | 62 |
| Complex  (≥3 cytogenetic abnormalities) | 2 | 143 |
